# Atezolizumab plus platinum-based chemotherapy as first-line therapy for metastatic urothelial cancer: A cost-effectiveness analysis

**DOI:** 10.3389/fphar.2022.872196

**Published:** 2022-08-22

**Authors:** Xiaoyan Liu, Yitian Lang, Qingqing Chai, Yan Lin, Yahui Liao, Yizhun Zhu

**Affiliations:** ^1^ State Key Laboratory of Quality Research in Chinese Medicine, School of Pharmacy, Macau University of Science and Technology, Taipa, Macau SAR, China; ^2^ Department of Pharmacy, Huangpu Branch, Shanghai Ninth People’s Hospital, Shanghai Jiao Tong University School of Medicine, Shanghai, China

**Keywords:** atezolizumab, cost-effectiveness, partitioned survival model, metastatic urothelial cancer, the perspective of the United States and China

## Abstract

**Purpose:** According to the IMvigor130 trial, adding atezolizumab to platinum-based chemotherapy was effective in the treatment of metastatic urothelial cancer (mUC). Based on the perspective of the United States and China, the current study evaluated cost-effectiveness of atezolizumab plus chemotherapy for mUC patients in the first-line setting.

**Methods:** A partitioned survival model was adopted for mUC patients. The survival data were derived from the IMvigor130 trial. Direct cost values were collected from the Centers for Medicare and Medicaid Services (CMS), Chinese Drug Bidding Database, and published literatures. The utility and toxicity data were gathered from related research studies and IMvigor130 trial. The incremental cost–utility ratios (ICURs) and incremental cost-effectiveness ratios (ICERs) were calculated and analyzed. Scenario analyses and sensitivity analyses were performed to observe the outputs and uncertainties.

**Results:** The base-case analysis showed that the ICUR of atezolizumab plus chemotherapy versus chemotherapy in American and Chinese settings is $ 737,371 /QALY and $ 385,384 /QALY, respectively. One-way sensitivity analyses showed that the ICUR ranged from $ 555,372/QALY to $ 828,205/QALY for the United States. Also, the range was from $ 303,099/QALY to $ 433,849/QALY in the Chinese setting. A probabilistic sensitivity analysis showed the likelihood that atezolizumab plus chemotherapy becoming the preferred strategy was a little low even if the price reduction strategy was applied.

**Conclusion:** Adding atezolizumab to chemotherapy improved survival time, but it is not a cost-saving option compared to chemotherapy for metastatic urothelial cancer patients in the American and Chinese settings.

## Introduction

Globally, bladder cancer is the 10th most common cancer, with 573,000 new cases and 213,000 deaths estimated in 2020 (Sung et al., 2021). Urothelial cancer is the most common type of bladder cancer, accounting for 90%–95% of all cases (Chen et al., 2020; Ren et al., 2020). Early-stage urothelial cancer is curable, but invasive urothelial cancer with progressive or recurrent disease usually has a poor prognosis (Lopez-Beltran et al., 2021). Patients with metastatic urothelial carcinoma (mUC), a chemotherapy-sensitive condition, typically receive platinum-based chemotherapy as their first course of treatment. A high proportion of patients who undergo such a treatment eventually develop platinum resistance and progressive diseases, even though the response rate is >50% (Holmsten et al., 2016). Nevertheless, many new regimens are currently being investigated because cytotoxic chemotherapy did not produce long-lasting results. A variety of cancers have responded to cancer immunotherapy in recent years. The mechanisms of action of all immunotherapies are the same: the agents engage the own immune system of the body to inhibit and kill cancer cells (Yang, 2015). In other words, immunotherapy is defined as a type of biotherapy that works by sensitizing the patient’s immune system to cancer, increasing selectivity to prevent immune escape (Akkın et al., 2021). The program death protein 1 (PD-1)/program death ligand 1 (PD-L1) axis is one key pathway that cancer cells use to avoid the body’s immune response. Many PD-1/PD-L1 blockers were produced to inhibit immune escape. Several clinical trials were conducted for mUC patients receiving PD-1/PD-L1 blockers. The KEYNOTE-045 trial showed that the median overall survival was 10.3 months with the pembrolizumab (A PD-1 inhibitor) group and 7.4 months with the chemotherapy group in the mUC patient (Bellmunt et al., 2017). The JAVELIN Bladder 100 trial indicated that adding avelumab (a PD-L1 inhibitor) to best supportive care prolonged the overall survival significantly in the mUC patients. The OS (overall survival) at 1 year was 79.1% and 60.4% in the avelumab group and control group, respectively (Powles et al., 2020). The IMvigor130 trial found that the addition of atezolizumab to chemotherapy prolonged PFS (progression-free survival) time (8.2 months vs. 6.3 months) and also improved the OS time (16 months vs. 13.4 months) compared with the chemotherapy group (Galsky et al., 2020). The results of these trials revealed that PFS/OS of mUC patients showed significant clinical improvement following treatment with PD-1/PD-L1 inhibitors. Based on these surprising results, some PD-1/PD-L1 inhibitors, such as atezolizumab, have been approved by the US Food and Drug Administration for urothelial cancer (US Food and Drug Administration, 2021). Several anti-neoplastic agents were concerned after approving, which might typically include concerns with increased prices and limited health gain (Cohen, 2017). Many health economic researchers have been thinking about why cancer occupies such a dominant position within healthcare systems across the world (Haycox, 2016). As a result, continual increases in expenditure on cancer medicines is continuing, which causes problems to healthcare systems across countries (Godman et al., 2021). To the best of our knowledge, there were few research studies that revealed the potential economic burden of mUC patients receiving atezolizumab. More evidence of economic studies and analyses to explore the economic burden of new anti-neoplastic drugs to decision makers or patients are very urgently needed. Although the IMvigor130 trial revealed a statistically significant PFS benefit in mUC patients receiving atezolizumab, the OS results did not cross the pre-specified threshold for significance. Whether the survival benefit reaches the expected value that matches the pricing needs to be further explored. Our study conducted the cost-effectiveness analysis of atezolizumab plus chemotherapy versus placebo plus chemotherapy to explore whether the current price is acceptable for mUC patients. Then, we conducted a comparative analysis from the perspectives of the United States and China because there is a large gap in threshold and affordability between middle-income and high-income countries, especially some drugs that have proved to be cost-effective in developed countries are not so cost-effective in developing countries (Al-Ziftawi et al., 2021). Also, investigating the differences of cost-effectiveness in atezolizumab plus chemotherapy for mUC between the US and China from the economic context is needed.

## Materials and methods

### Model structure

An analysis of the cost-effectiveness was conducted using a partitioned survival model (PSM) to simulate the disease survival states of mUC patients beyond the follow-up period of the clinical trial. The characteristics of included patients of the study were consistent with those of the IMvigor130 trial (Galsky et al., 2020), who were aged 18 years or older with locally advanced or metastatic urothelial carcinoma and had not received previous systemic therapy in the metastatic setting. One of two interventions is offered to patients in this study until disease progression occurs: (i) platinum-based chemotherapy (34% of patients received cisplatin with gemcitabine and 66% of patients received carboplatin with gemcitabine); (ii) atezolizumab plus platinum-based chemotherapy (30% of patients received cisplatin with atezolizumab and gemcitabine and 70% of patients received carboplatin with atezolizumab and gemcitabine). In case of disease progression, it is assumed that the current treatment regimen became invalid, and the initial regimen would be replaced by subsequent best supportive therapies for the patients with the progressed disease.

**FIGURE 1:**
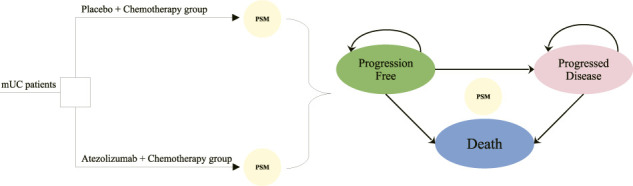
Model structure overview. mUC, metastatic urothelial cancer; PSM, partitioned survival model.

Three mutually exclusive disease health states were set in the partitioned survival model, including progression-free (PF) survival, progressed disease (PD), and death. The decision tree diagram and bubble diagram of the model are shown in Figure 1. The initial health state that mUC patients entered the model is the PF state, which is able to move to the PD or death state based on survival data. Patients were assumed to be unable to return to previous health states. In accordance with the IMvigor130 protocol, the period for the model cycle was 21 days. In order to fully understand the outcome of the disease, one needs to extrapolate limited survival data to predict long-term outcomes. The ten-year timeframe was therefore set to ensure that mUC patients fully transited to the terminal state.

### Clinical data

The available observational time of the IMvigor130 trial was around 30 months for OS and PFS. Also, extrapolating over follow-up time was needed in order to predict survival over a ten-year period. We used algorithms proposed by Guyot to get the simulated individual patient-level data (Guyot et al., 2012). Engauge Digitizer, a tool for digitizing pictures, is used to digitize the OS and PFS Kaplan–Meier curves for each treatment regimen. The generated individual patient-level data (IPD) were applied to fit a range of parametric distributions, including Weibull, exponential, Gompertz, log-logistic, and log-normal. In general, the most appropriate distribution is determined by the Akaike information criterion (AIC) (Kuk and Varadhan, 2013). The key clinical data are shown in Table 1.

**TABLE 1:** Projected survival data and safety data summary.

Parameter	Expected value	Range	Distribution	Reference
PFS: atezolizumab + chemotherapy	Shape = 1.7445; scale = 10.9865	1.5951–1.9078	Log-logistic	Galsky et al. (2020)
		9.9954–12.076		
PFS: placebo + chemotherapy	Shape = 2.0017; scale = 9.6519	1.8263–2.1939	Log-logistic	Galsky et al. (2020)
		8.8593–10.5154		
OS: atezolizumab + chemotherapy	Shape = 1.5267; scale = 23.5327	1.3676–1.7044	Log-logistic	Galsky et al. (2020)
		20.9135–26.48		
OS: placebo + chemotherapy	Shape = 1.6106; scale = 20.2234	1.4404–1.801	Log-logistic	Galsky et al. (2020)
		18.0261–22.6886		
Probability of main grade 3 or 4 adverse events in the atezolizumab + chemotherapy arm				
Neutrophil count decreased	5.3%	4.0%–6.6%	Beta	Galsky et al. (2020)
Anemia	8.2%	6.1%–10.3%	Beta	Galsky et al. (2020)
Neutropenia	8.4%	6.3%–10.5%	Beta	Galsky et al. (2020)
Thrombocytopenia	4.0%	3.0%–5.0%	Beta	Galsky et al. (2020)
Probability of main grade 3 or 4 adverse events in the placebo + chemotherapy arm				
Neutrophil count decreased	6.4%	4.8%–8.0%	Beta	Galsky et al. (2020)
Anemia	7.4%	5.6%–9.3%	Beta	Galsky et al. (2020)
Neutropenia	4.4%	3.3%–5.5%	Beta	Galsky et al. (2020)
Thrombocytopenia	3.6%	2.7%–4.5%	Beta	Galsky et al. (2020)

PFS, progression-free survival; OS, overall survival; AIC, Akaike information criterion.

### Costs and utilities

This analysis adopted the perspective of the health sector with different settings of the United States and China. The direct medical costs that were considered are as follows: agent acquisition costs, administration costs for intravenous injection, management of adverse events (AEs), and palliative care. The doses of agents are kept with those of the IMvigor130 trial. In the platinum-based chemotherapy regimen, gemcitabine was used at a dose of 1,000 mg/m^2^ body surface area (BSA) administered intravenously on days 1 and 8 of each model cycle. Carboplatin (area under the curve of 4.5 mg/ml per min administered intravenously) or cisplatin (70 mg/m^2^ BSA administered intravenously) was administered on day 1 of each cycle. In the atezolizumab plus chemotherapy regimen, the doses of chemotherapy agents are adopted in keeping with the aforementioned chemotherapy regimen, and atezolizumab was administered at a dose of 1,200 mg on day 1 of each cycle. In this analysis, the mean BSA of 1.85 m^2^ is adopted for American patients (Slater et al., 2020), and that of Chinese patients is 1.72 m^2^ (Lu et al., 2017). The prices of gemcitabine, carboplatin, cisplatin, and atezolizumab in the US were sourced from the Centers for Medicare and Medicaid Services (CMS) (Centers for Medicare&Medicaid Services, 2022), and those of China were acquired from drug acquisition costs in a local charge database (Yaozh, 2022). Costs related to administration cost for intravenous injection, palliative care, and best supportive care (BSC) were derived from CMS or related articles for analysis in an American setting (Centers for Medicare and Medicaid Services, 2022; Wu et al., 2018; Aly et al.,2919), and the cost data for analysis in the Chinese setting were gathered from published literatures (Liu et al., 2021). The IMvigor130 trial shared data about incidences of adverse events. It was assumed that AEs of grades 1 and 2 could be well managed, and the costs of that were not included. So, only the management costs of grade 3 or 4 AEs were considered. The data about costs of managing AEs were sourced from open-accessed databases or published literatures (Liu et al., 2021; Agency for Healthcare Research and Quality, 2022; Lang et al., 2020). As the IMvigor130 trial reported, around 26% of patients in the atezolizumab plus chemotherapy group and 41% of those in the chemotherapy group receive subsequent therapies. The proportions corresponded to the baseline data and were only used for cost estimates. All costs reported for years prior to 2021 are updated to December 2021 in US dollars (USD) using the Consumer Price Index (CPI). All costs sourced from China in this study were converted into US dollars based on the average exchange rate from January to October 2021. More details about costs are summarized in Table 2.

**TABLE 2:** Model costs, utility estimates, and other parameters.

Parameter	Distribution	US		China		
**Treatment cost**		**Value (range), USD**	**Reference**	**Value (range), USD**		**Reference**
Atezolizumab (per 1,200 mg)	Gamma	9,569.88 (7,177–9,569.88)	Centers for Medicare&Medicaid Services, (2022)	5,073.55 (3,805.16–5,073.55)		Yaozh, (2022)
Gemcitabine (per 200 mg)	Gamma	70.98 (53.24–88.73)	Centers for Medicare&Medicaid Services, (2022)	7.17 (5.38–8.96)		Yaozh, (2022)
Cisplatin (per 10 mg)	Gamma	1.77 (1.33–2.21)	Centers for Medicare&Medicaid Services, (2022)	1.17 (0.88–1.46)		Yaozh, (2022)
Carboplatin (per 50 mg)	Gamma	2.643 (1.98–3.30)	Centers for Medicare&Medicaid Services, (2022)	12.22 (9.17–15.28)		Yaozh, (2022)
Administration (per cycle)	Gamma	399.88 (299.91–499.85)	Centers for Medicare and Medicaid Services, (2022)	61.72 (46.29–77.15)		Liu et al. (2021)
Best supportive care (per cycle)	Gamma	6,199.62 (4,649.72–7,749.53)	Aly et al. (2019)	1,415.02 (1,061.27–1,768.78)		Liu et al. (2021)
Terminal care	Gamma	11,820 (8,865–14,775)	Wu et al. (2018)	2,099.15 (1,574.36–2,623.94)		Liu et al. (2021)
**AE unit costs**		**Value (range), USD [in 2015, USD]**	**Reference**	**Value (range), USD**		**Reference**
Neutrophil count decreased	Gamma	51,308 (38,481–64,135) [43,707]	Agency for Healthcare Research and Quality, (2022)	104.95 (78.71–131.19)		Liu et al. (2021)
Neutropenia	Gamma	51,337 (38,503–64,171) [43,732]	Agency for Healthcare Research and Quality, (2022)	526.90 (395.18–658.63)		Liu et al. (2021)
Anemia	Gamma	36,264 (27,198–45,330) [30,892]	Agency for Healthcare Research and Quality, (2022)	607.06 (455.30–758.83)		Liu et al. (2021)
Thrombocytopenia	Gamma	45,332 (33,999–56,665) [38,617]	Agency for Healthcare Research and Quality, (2022)	4,082.99 (3,062.24–5,103.74)		Lang et al. (2020)
**Utility estimate**		**Value (range)**		**Reference**		
Progression-free disease	Beta	0.80 (0.77–0.82)		Hale et al. (2021)		
Progressive disease	Beta	0.75 (0.70–0.79)		Hale et al. (2021)		
**Other parameter**		**Value (range)**	**Reference**	**Value (range)**		**Reference**
Body surface area, m^2^	Normal	1.85 (1.49–2.21)	Slater et al. (2020)	1.72 (1.50–1.90)	Lu et al. (2017)	
**Proportion of cisplatin in the initial treatment regimen**		**Value (range)**		**Reference**		
AC group	Beta	30% (22.5%–37.5%)		Galsky et al. (2020)		
PC group	Beta	34% (25.5%–42.5%)		Galsky et al. (2020)		
**Proportion of carboplatin in the initial treatment regimen**		**Value (range)**		**Reference**		
AC group	Beta	70% (62.5%–77.5%)		Galsky et al. (2020)		
PC group	Beta	66% (57.5%–74.5%)		Galsky et al. (2020)		
**Proportion of patients receiving subsequent therapy**		**Value (range)**		**Reference**		
AC group	Beta	26% (19.5%–32.5%)		Galsky et al. (2020)		
PC group	Beta	41% (30.8%–51.3%)		Galsky et al. (2020)		

The costs of AEs presented in this table were paid on a per-event basis. All costs reported for years prior to 2021 are updated to December 2021 USD using the American and Chinese CPI. All costs sourced from China in this study were converted into US dollars ($1 = RMB 6.4649, average exchange rate from January to October 2021).

AC, atezolizumab plus chemotherapy; PC, placebo plus chemotherapy; AEs, adverse events; CPI, Consumer Price Index; USD, US dollars; CMS, Centers for Medicare and Medicaid Services.

In this partitioned survival model, each health state was assigned a health utility value based on the disease progression context. Since the data from the EuroQol 5-Dimension (EQ-5D) in the IMvigor130 trial would not be reported in their clinical study report, the direct quality of life data could not be available. Highly relevant and robust data are extremely crucial. Since the quality of life is related to the progressive stage, the utility estimates for PF and PD states were assumed to be 0.80 and 0.75, respectively, based on similar UC studies (Hale et al., 2021).

### Analyses

In the base-case analysis, we used incremental cost-effectiveness ratios (ICERs) to evaluate the incremental cost per additional life-year (LY) gained between atezolizumab plus chemotherapy and placebo plus chemotherapy regimens. Incremental cost–utility ratios (ICURs) were used to assess the incremental cost per additional quality-adjusted life-year (QALY). All QALYs and costs were discounted at an annual rate of 3% for the United States, and 5% was adopted for China. If the ICUR of atezolizumab plus chemotherapy compared with placebo plus chemotherapy is below the willingness-to-pay (WTP) threshold, the atezolizumab plus chemotherapy regimen is regarded as a cost-effective option. The threshold for WTP in the United States is usually in the range of approximately $ 100,000–150,000/QALY (Verma et al., 2018). In this analysis, we adopted $ 100,000/QALY as the WTP threshold for the cost-effectiveness analysis in the setting of the United States. In China, the WTP threshold was set at thrice the per capita gross domestic product (GDP, calculated to be $31,316 in 2020) (Hutubessy et al., 2003).

We conducted one-way and probabilistic sensitivity analyses (PSA) for model input parameters in order to assess the robustness of our results and to identify the variables that had a considerable impact on them. In one-way sensitivity analyses, the range of the discount rate is from 0 to 8 %, and other inputs were assumed a variation by ± 25% of the base-case value. In addition, Monte Carlo simulation of 1,000 iterations was used to run the PSA. According to specific probability distributions, all input parameters were sampled simultaneously. Health utilities and incidence of adverse events or proportions were sampled from beta distribution and gamma distribution for costs (Briggs et al., 2012). A cost-effectiveness acceptability curve (CEAC) was generated to clearly present the likelihood that atezolizumab plus chemotherapy was cost-effective at a range of WTP threshold. The partitioned survival model and cost-effectiveness analysis model were created and programmed in R (version 4.1.2, http://www.r-project.org).

## Results

### Validity of the fitted parametric survival function

**FIGURE 2:**
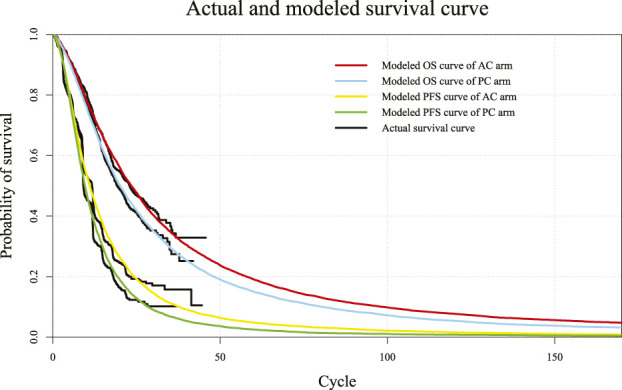
Diagram of modeled PFS and OS fit curves in different regimens. The colored lines represent the modeled survival curves, and the black lines represent the actual survival curves. Each cycle of the x-axis is 3 weeks. PFS, progression-free survival; OS, overall survival; AC, atezolizumab plus chemotherapy; PC, placebo plus chemotherapy.

Log-logistic-predicted PFS and OS models of atezolizumab plus chemotherapy and placebo plus chemotherapy regimens and actual survival curves are shown in Figure 2. The selected distribution of the projected curve is shown in Table 1. All detailed values of the parametric distributions for each arm are listed in Supplementary Table S1.

### Base-case analysis

In the US context, patients with mUC receiving the atezolizumab plus chemotherapy regimen gained 2.290 LYG, 1.651 QALYs, and expended $ 233,492, and patients receiving the placebo plus chemotherapy regimen resulted in 1.957 LY, 1.419 QALYs gained, and $ 62,422 expended. Compared with the placebo plus chemotherapy regimen, the atezolizumab plus chemotherapy regimen increased the overall cost by $ 171,070. For effectiveness, the atezolizumab plus chemotherapy regimen showed an increase of 0.333 LYG and 0.232 QALYs compared with the placebo plus chemotherapy regimen. The results of the average cost-effectiveness ratios of atezolizumab plus chemotherapy are $ 101,962 /LY and $ 141,425 /QALY, and those of the placebo plus chemotherapy regimen are $ 27,259 /LY and $ 37,809 /QALY, respectively. The ICER and ICUR of atezolizumab plus chemotherapy compared with placebo plus chemotherapy are $ 513,724 /LY and $ 737,371 /QALY, respectively.

In the context of China, patients with mUC receiving the atezolizumab plus chemotherapy regimen gained 2.290 LYG, 1.580 QALYs, and expended $ 96,946, and patients receiving the placebo plus chemotherapy regimen resulted in 1.957 LY, 1.365 QALYs gained, and $ 9,912 expended. Compared with the placebo plus chemotherapy regimen, the atezolizumab plus chemotherapy regimen increased the overall cost by $ 87,034. For effectiveness, the atezolizumab plus chemotherapy regimen showed an increase of 0.333 LYG and 0.215 QALYs compared with the placebo plus chemotherapy regimen. The results of the average cost-effectiveness ratios of atezolizumab plus chemotherapy are $ 42,334 /LY and $ 61,358 /QALY, and those of the placebo plus chemotherapy regimen are $ 5,065 /LY and $ 7,262 /QALY, respectively. The ICER and ICUR of atezolizumab plus chemotherapy compared with placebo plus chemotherapy are $ 261,363 /LY and $ 404,809 /QALY, respectively. All results of the base-case analysis for the United States and China are summarized in Table 3.

**TABLE 3:** Results of the base-case analysis and subgroup analysis.

Country	Regimen	LY	QALY	Cost, US$	ICER ($/LY)	ICUR ($/QALY)
US	Placebo plus chemotherapy	1.957	1.419	62,422	-	-
	Atezolizumab plus chemotherapy	2.290	1.651	233,492	513,724	737,371
China	Placebo plus chemotherapy	1.957	1.365	9,912	-	-
	Atezolizumab plus chemotherapy	2.290	1.580	96,946	261,363	404,809

LY, life-year; QALY, quality-adjusted life-year; ICER, incremental cost-effectiveness ratio; ICUR, incremental cost–utility ratio.

### One-way sensitivity analysis

**FIGURE 3:**
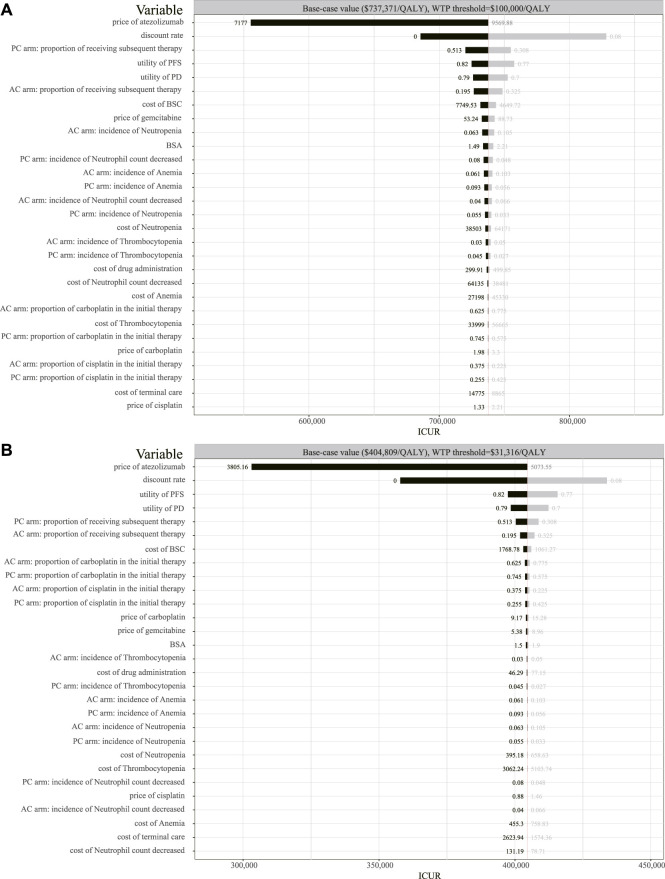
Tornado diagram of the one-way sensitivity analysis. **(A)** Output in the American setting. **(B)** Output in the Chinese setting. QALY, quality-adjusted life-year; BSC, best supportive care; BSA, body surface area; ICUR, incremental cost–utility ratio; AC, atezolizumab plus chemotherapy; PC, placebo plus chemotherapy.

The one-way sensitivity analyses were conducted to test the modeling assumptions. The results are shown in the form of tornado diagrams (Figure 3). In the setting of the United States, the tornado diagram showed that the price of atezolizumab and the discount rate were the top two variables that have a significant impact on ICUR. Also, the proportion of receiving subsequent therapy for the placebo plus chemotherapy group ranked third in the tornado diagram. In addition, the higher this proportion is, the lower the value of ICUR is. The utility of PFS, utility of PD, and the proportion of receiving subsequent therapy for the atezolizumab plus chemotherapy group also have a significant impact on ICUR. The result of the one-way sensitivity analysis ranged from $ 555,372/QALY to $ 828,205/QALY for the United States. The impact of the AE-related, BSC-related, or palliative-related expenditure on the outcome was minimal. Similar to the results of the American setting, the one-way sensitivity analysis for China revealed that the top-ranked variables are still the price of atezolizumab, discount rate, the utility of PFS, the utility of PD, and the proportion of receiving subsequent therapy. The range for the one-way sensitivity analysis was from $ 303,099/QALY to $ 433,849/QALY in the Chinese setting. Either in the United States or China, reducing the price of atezolizumab contributes the most to the reduction of the ICUR value.

### Probabilistic sensitivity analysis

**FIGURE 4:**
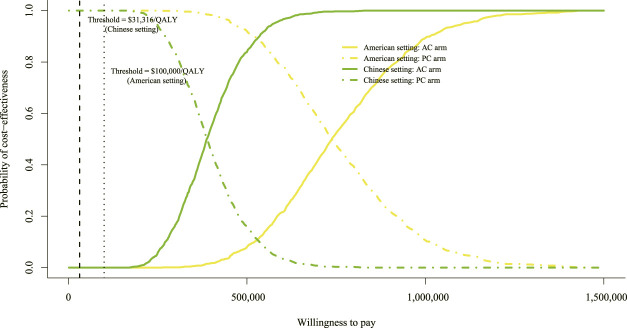
Cost-effectiveness acceptable curve. The y-axis indicates the probability that a regimen is cost-effective across the willingness-to-pay threshold (x-axis). QALY, quality-adjusted life-year; AC, atezolizumab plus chemotherapy; PC, placebo plus chemotherapy.

A total of 1,000 iterations were conducted to sample all the model parameters from probability distributions simultaneously. To assess whether atezolizumab plus chemotherapy would be considered cost-effective at various levels of WTP in terms of health gains, we designed a CEAC (Figure 4). Either in the setting of the United States or China, the CEAC revealed a zero probability of adding atezolizumab to chemotherapy being cost-effective. As the tornado diagram indicated that the price of atezolizumab contributes the most to the reduction of the ICUR value, additional probabilistic sensitivity analyses of adjusting the price of atezolizumab to 75%, 50%, and 25% of its price were conducted. Also, two scenarios of the WTP threshold were analyzed for the United States ($ 100,000/QALY and $ 200,000/QALY) and China ($ 31,316/QALY and $ 60,000/QALY).

In the context of the United States, if the WTP threshold was $ 100,000/QALY, the likelihood of atezolizumab plus chemotherapy in the price reduction setting was 0%, 0%, and 3.2% of being cost-effective, respectively (settings of 25%, 50%, and 75% reduction in the price of atezolizumab). When the threshold of $ 200,000/QALY was adopted, the likelihood of atezolizumab plus chemotherapy was 0%, 2.1%, and 54.8%, respectively. Also, in the setting of China, if the WTP threshold was $ 31,316/QALY, the likelihood of atezolizumab plus chemotherapy was 0%, 0%, and 0.1%, respectively. When the threshold of $ 60,000/QALY was used, the likelihood of atezolizumab plus chemotherapy was 0%, 0%, and 5.3%, respectively. The CEAC of price reduction assumption is shown in Supplementary Figure S1. The probability of a regimen becoming the preferred strategy is summarized in Supplementary Table S2.

## Discussion

The durable activity and good tolerability of atezolizumab for urothelial cancer were reported based on a clinical trial of phase II (Rosenberg et al., 2016). It provided a new therapy choice for mUC patients, and the FDA issued an accelerated approval for atezolizumab in the second-line treatment of urothelial cancer in 2016. A study revealed that the agent brought a significant economic burden (Savage, 2017). However, recent reports about the clinical benefits of atezolizumab plus chemotherapy for mUC patients based on a clinical trial of phase III sparked great interest among both oncologists and patients (Galsky et al., 2020). This evaluation explored the cost-effectiveness of adding atezolizumab to platinum-based chemotherapy based on the latest survival data in the American and Chinese settings. The base-case analysis results showed that the ICUR of atezolizumab plus chemotherapy compared with chemotherapy alone is $ 737,371/QALY in the American setting and $ 404,809/QALY in the Chinese context. The ICUR values sharply exceed the average threshold of $ 100,000/QALY in the United States and $ 31,316/QALY in China. Our one-way sensitivity showed that the variable that made the greatest impact on ICUR was the price of atezolizumab. To further investigate whether the lower price of atezolizumab or in the setting of high-income regions or cities could make this regimen become cost-effective, we made following assumptions: (i) the price of atezolizumab was cut by 25%, 50%, and 75%; (ii) the WTP threshold was increased to $ 200,000/QALY in the American setting and $ 60,000/QALY in the Chinese setting. The additional CEAC showed that at the threshold of $ 100,000/QALY, even if the price of atezolizumab is reduced by 75%, it is only 3.2% of the likelihood to be cost-effective in the American context. Under the same premise, the probability of atezolizumab plus chemotherapy is around 54.8% at a $ 200,000/QALY threshold. In the context of China, even if the price of atezolizumab is cut by 75%, it is only 0.1% of probability of being cost-effective at the threshold of $ 31,316/QALY. Under the same assumption, the probability of atezolizumab plus chemotherapy is around 5.3 % at the $ 60,000/QALY threshold.

Our additional analysis revealed that although the price of atezolizumab plays a key role, lowering the price of atezolizumab does not improve the likelihood of becoming cost-effective significantly. Also, the QALY gained in the atezolizumab plus chemotherapy group just exceeded 0.23 QALY compared with the placebo plus chemotherapy group. Therefore, the atezolizumab regimen hardly became a cost-effective treatment choice for patients and oncologists. We noted that similar economic studies on pembrolizumab for urothelial cancer showed an improvement in survival benefit significantly. Hale et al. (2021) concluded that pembrolizumab was a cost-effective alternative to chemotherapy based on a US third-party healthcare payer’s perspective, with a significant QALY benefit (2.91 QALYs in the pembrolizumab group vs. 0.90 QALYs in the chemotherapy group). Similarly, Patterson et al. (2019) concluded that pembrolizumab was a cost-effective choice compared to chemotherapy from a Swedish healthcare perspective. The QALY of Patterson’s study was 2.93 QALYs and 0.82 QALYs in pembrolizumab and chemotherapy groups, respectively. We found that, also as immune checkpoint inhibitors, differences in survival time between PD-1 inhibitors and PD-L1 inhibitors were significant for urothelial cancer under a similar premise. Pembrolizumab, a PD-1 blocker, improved the OS time and QALYs significantly, and its price matched its survival improvement. But the OS improvement of adding atezolizumab to chemotherapy was not statistically significant, and the price of atezolizumab exceeded the value that matches its survival improvement. In other words, the significant improvement of OS is also important; only the significant improvement of PFS contributes little to the economic results of drugs. This might be a major reason why atezolizumab plus chemotherapy was not a cost-effective alternative.

Some weakness existed in our study. First, our survival data were derived from IMvigor130, in which around three-fourths of the patients were white. Asian patients accounted for around one-fifth. However, our survival analysis was based on the overall patients whether in the American or Chinese setting. Inevitably, the accuracy of survival data was slightly shaken by race. Second, our study relied on modeling techniques. It was not an actual IPD in this model, but a projected IPD generated by a specific algorithm. Third, the analysis results using parametric models to extrapolate the survival outcomes beyond the time horizon may result in a slight hypothesis bias compared to the analysis results with sufficient survival data of the follow-up. Although it could undermine the robustness, the sensitivity analyses covered the substantial ranges of all variables in order not to ignore the uncertainties. By using modeling techniques, it is possible to predict certain changes in the results. Finally, the data about quality of life, sourced from the IMvigor130 trial, were not reported. Direct health-associated utility data were not available. Thus, we can only extract utility data from published literatures. However, our sensitivity analysis shows that the change in utility did not have a significant impact on ICUR. Furthermore, our analysis had one notable feature in addition to its limitations. As we selected models, we put in a great deal of effort. In this study, we considered the Markov model, partitioned survival model, and cure model. Considering the characteristics of the survival curve, the cure model was not adopted. Likewise, in order to reduce the deviation caused by the hypothesis, we ultimately chose the partitioned survival model over the Markov model. By using the partitioned survival model, it is possible to obtain the survival cohort proportion directly from the survival curve, thereby reducing the hypothesis bias in calculating the transition probability from PF or PD to death.

It is hoped that this analysis can provide help to clinicians, health decision-makers, and patients. More studies of this kind are also expected to be published as evidence is updated to continuously improve credibility of this economic evaluation.

## Conclusion

Patients with metastatic urothelial cancer following treatment with atezolizumab plus chemotherapy showed more survival benefits than those with the placebo plus chemotherapy regimen. Although the economic gap between the United States and China is obvious, the conclusions of the cost-effectiveness analysis are consistent. Our economic evaluation concluded that the addition of atezolizumab to chemotherapy is not cost-effective compared with the chemotherapy regimen at a $ 100,000/QALY threshold in the United States. The conclusion is also applicable in the context of China. Adding atezolizumab to chemotherapy compared with chemotherapy alone is not cost-effective in the threshold of a $ 31,316/QALY setting.

## Data Availability

The original contributions presented in the study are included in the article/Supplementary Material; further inquiries can be directed to the corresponding author.
