# Supplementary material for: Atezolizumab plus platinum-based chemotherapy as first-line therapy for metastatic urothelial cancer: A cost-effectiveness analysis
Source: Front Pharmacol. 2022 Aug 22;13:872196. doi: 10.3389/fphar.2022.872196 (PMC9441572; doi:10.3389/fphar.2022.872196)
Supplement: Supplementary file 1 [file DataSheet1.PDF]

Table S1. The values of parametric model for each arm.

**Arm**	Survival Curve	Distribution	Values (95% CI)	AIC
Atezolizumab plus chemotherapy	OS	Log-logistic	shape: 1.53 (1.37 to 1.7), scale: 23.53 (20.91 to 26)	2122.54
		Weibull	shape: 1.24 (1.11 to 1.3), scale: 32.60 (29.21 to 36)	2130.134
		Exponential	rate: 0.03 (0.02 to 0.0)	2141.315
		Log-normal	meanlog: 3.18 (3.05 to 3.3), sdlog: 1.20 (1.09 to 1.3)	2131.537
		Gompertz	shape: 0.01 (0.00 to 0.0), rate: 0.02 (0.02 to 0.0)	2140.016
	PFS	Log-logistic	shape: 1.74 (1.60 to 1.9), scale: 10.99 (10.00 to 12)	2493.742
		Weibull	shape: 1.22 (1.12 to 1.3), scale: 16.32 (14.95 to 17)	2525.386
		Exponential	rate: 0.06 (0.06 to 0.0)	2541.96
		Log-normal	meanlog: 2.39 (2.29 to 2.4), sdlog: 1.02 (0.94 to 1.1)	2502.719
		Gompertz	shape: 0.01 (0.01 to 0.0), rate: 0.06 (0.05 to 0.0)	2543.34
Placebo plus chemotherapy	OS	Log-logistic	shape: 1.61 (1.44 to 1.8), scale: 20.22 (18.03 to 22)	1973.662
		Weibull	shape: 1.29 (1.15 to 1.4), scale: 27.87 (25.09 to 30)	1977.645
		Exponential	rate: 0.03 (0.03 to 0.0)	1993.089
		Log-normal	meanlog: 3.02 (2.89 to 3.1), sdlog: 1.15 (1.04 to 1.2)	1987.741
		Gompertz	shape: 0.02 (0.01 to 0.0), rate: 0.03 (0.02 to 0.0)	1987.919
	PFS	Log-logistic	shape: 2.00 (1.83 to 2.1), scale: 9.65 (8.86 to 10.)	2272.182
		Weibull	shape: 1.37 (1.25 to 1.4), scale: 13.59 (12.54 to 14)	2293.078
		Exponential	rate: 0.08 (0.07 to 0.0)	2333.96
		Log-normal	meanlog: 2.24 (2.15 to 2.3), sdlog: 0.92 (0.85 to 0.9)	2293.324
		Gompertz	shape: 0.03 (0.01 to 0.0), rate: 0.06 (0.05 to 0.0)	2324.848

AIC Akaike information criterion, *CI* Confidence interval, *OS* Overall survival, *PFS* Progression free survival.

Table S2. The probability of therapy regimen becoming cost-effective with the reduction of price of atezolizumab.

**Country**	Threshold	Regimen	The likelihood of cost-effective when the price of the atezolizumab decreased by _________			
			0%	25%	50%	75%
**The United States**	$100,000/QALY	PC	100%	100%	100%	96.8%
		AC	0	0%	0%	3.2%
	$200,000/QALY	PC	100%	100%	97.9%	45.2%
		AC	0	0%	2.1%	54.8%
**China**	$31,316/QALY	PC	100%	100%	100%	99.9%
		AC	0	0%	0%	0.1%
	$60,000/QALY	PC	100%	100%	100%	94.7%
		AC	0	0%	0%	5.3%

PC placebo plus chemotherapy, ***AC*** atezolizumab plus chemotherapy, ***QALY*** quality-adjusted life-year.

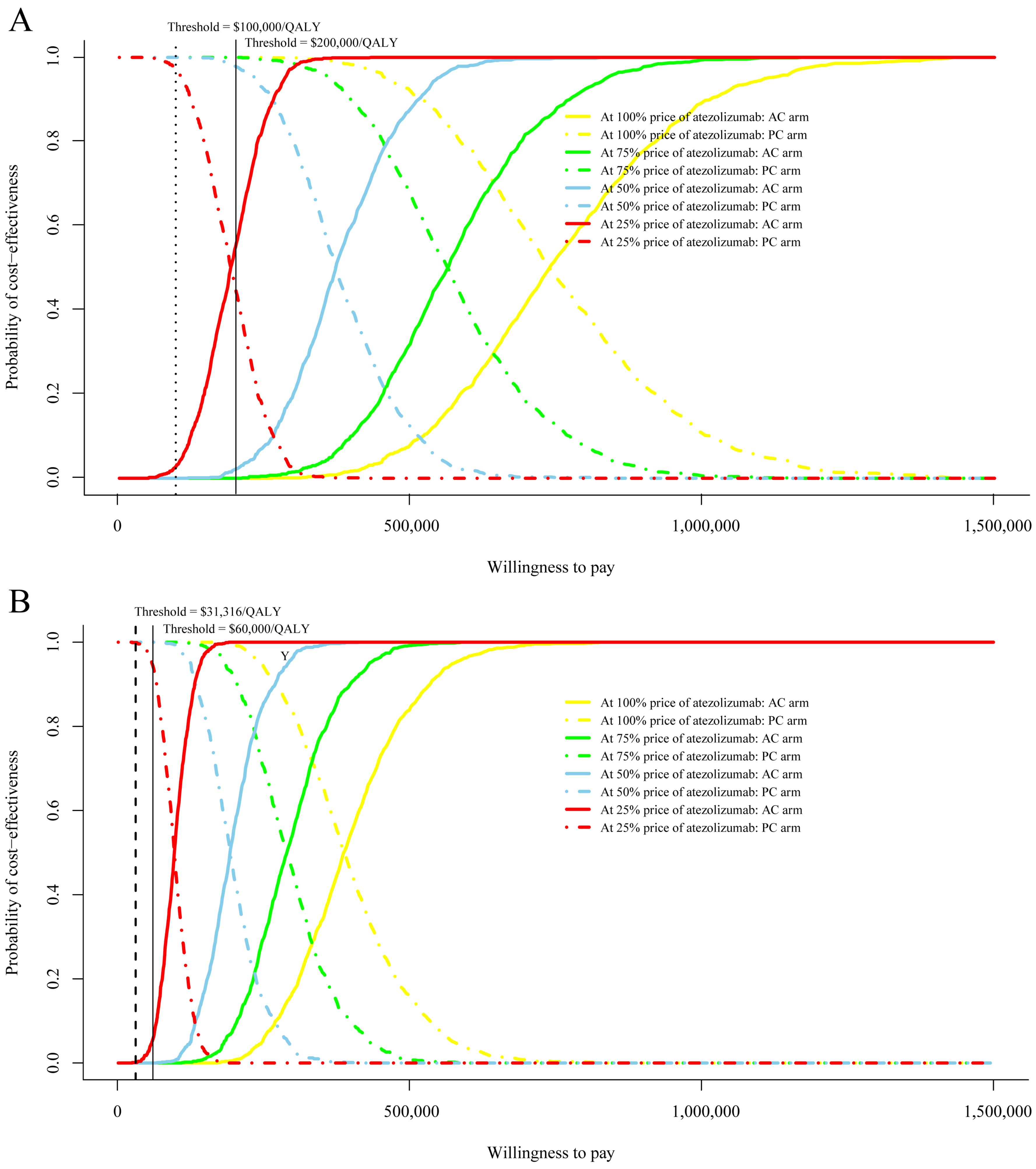

Figure S1. Cost-effectiveness acceptable curve. (A)The output in the American setting. (B) The output in the Chinese setting. The y-axis indicates the probability that a regimen is cost-effective across the willingness-to-pay threshold (x-axis). ***QALY*** quality-adjusted life-year, ***AC*** atezolizumab plus chemotherapy, ***PC*** placebo plus chemotherapy.
